# Asian patients’ perspectives on advance care planning: A mixed-method systematic review and conceptual framework

**DOI:** 10.1177/02692163211042530

**Published:** 2021-09-06

**Authors:** Diah Martina, Olaf P Geerse, Cheng-Pei Lin, Martina S Kristanti, Wichor M Bramer, Masanori Mori, Ida J Korfage, Agnes van der Heide, Judith AC Rietjens, Carin CD van der Rijt

**Affiliations:** 1 Department of Medical Oncology, Erasmus MC Cancer Institute, University Medical Center Rotterdam, Rotterdam, The Netherlands; 2 Department of Public Health, Erasmus MC, University Medical Center Rotterdam, Rotterdam, The Netherlands; 3 Division of Psychosomatic and Palliative Medicine, Department of Internal Medicine, Universitas Indonesia, Jakarta, Indonesia; 4 Dr. Cipto Mangunkusumo National Center Hospital, Jakarta, Indonesia; 5 Department of Internal Medicine, Amsterdam University Medical Center, Amsterdam, The Netherlands; 6 Institute of Community Health Care, School of Nursing, National Yang Ming Chiao Tung University, Taipei; 7 Florence Nightingale Faculty of Nursing, Midwifery and Palliative Care, Cicely Saunders Institute of Palliative Care, Policy and Rehabilitation, King’s College London, London, UK; 8 School of Nursing, Faculty of Medicine, Public Health and Nursing, Universitas Gadjah Mada, Yogyakarta, Indonesia; 9 Medical Library, Erasmus MC, University Medical Center Rotterdam, Rotterdam, The Netherlands; 10 Palliative and Supportive Care Division, Seirei Mikatahara General Hospital, Hamamatsu, Japan

**Keywords:** Asian continental ancestry group, critical illness, attitude, patient preference, mixed design, systematic review

## Abstract

**Background:**

Asian healthcare professionals hold that patients’ families play an essential role in advance care planning.

**Aim:**

To systematically synthesize evidence regarding Asian patients’ perspectives on advance care planning and their underlying motives.

**Design:**

Mixed-method systematic review and the development of a conceptual framework (PROSPERO: CRD42018099980).

**Data sources:**

EMBASE, MEDLINE, Web of Science, and Google Scholar were searched for studies published until July 27, 2020. We included studies concerning seriously-ill Asian patients’ perspectives on advance care planning or their underlying motives for engaging or not engaging in it.

**Results:**

Thirty-six articles were included; 22 were quantitative and 27 were from high-income countries. Thirty-nine to ninety percent of Asian patients were willing to engage in advance care planning. Our framework highlighted that this willingness was influenced not only by their knowledge of their disease and of advance care planning, but also by their beliefs regarding: (1) its consequences; (2) whether its concept was in accordance with their faith and their families’ or physicians’ wishes; and (3) the presence of its barriers. Essential considerations of patients’ engagement were their preferences: (1) for being actively engaged or, alternatively, for delegating autonomy to others; (2) the timing, and (3) whether or not the conversations would be documented.

**Conclusion:**

The essential first step to engaging patients in advance care planning is to educate them on it and on their diseases. Asian patients’ various beliefs about advance care planning should be accommodated, especially their preferences regarding their role in it, its timing, and its documentation.

**What is already known about the topic?**

- Asian healthcare professionals hold that patients’ family play a central role in advance care planning and rarely engage patients in it.
- Despite the wide range of studies on advance care planning in different populations in Asian countries, and despite their variety of methodologies and conceptualizations of advance care planning, there has been no systematic synthesis of their results.

**What this papers adds**

- This study demonstrates that although a majority of Asian patients regarded advance care planning as necessary, more varied results were produced by studies that examined their actual willingness to engage in it.
- Willingness to engage in advance care planning was affected not only by patients’ knowledge of their disease and advance care planning, but also by their beliefs: (a) about its advantages or disadvantages; (b) that its concept should be in accordance with patients’ faith and their families’ or physicians’ wishes; and (c) about the presence of barriers to it (e.g. complexities of future planning, socioeconomic dependence, and the unreadiness of the healthcare system).

**Implications for practice, theory, or policy**

- Initial steps toward engaging Asian patients in advance care planning should include: (a) an exploration of their understanding of their disease; and (b) the correction of common misperceptions through education on what advance care planning entails.
- Advance care planning for Asian patients needs to accommodate: (a) patients’ widely differing beliefs on it; (b) their preferences regarding the way in which values are communicated, that is, when and by whom; and (c) whether or not it is documented.

## Introduction

The implementation of advance care planning has become one of the indicators for high-quality palliative care.^1^ Advance care planning enables patients to define, discuss, and record their goals and preferences for future medical treatment and care, and to review these preferences if appropriate.^2^ It also aims to clarify and document patients’ values and preferences regarding future medical care, and to ensure these are taken into account at the time of incapacity.^2^ To ensure that these values and preferences are acknowledged and can be used to facilitate respectful and responsive care, patients’ involvement in this process is deemed essential.^3^

The practice of advance care planning may be affected by societal norms and values.^4,5^ In our systematic review of Asian healthcare professionals’ perspectives on advance care planning, we found that professionals regard families as playing the leading role in it.^6^ However, we also observed that these professionals rarely engage patients in advance care planning, even when the patients retain their decision-making capacity. Among the reasons for not engaging patients was healthcare professionals’ concern about patients’ lack of readiness to engage in advance care planning.^6^

To better understand how advance care planning can best be delivered to Asian patients, it is essential to understand their preferences. Although various studies have been conducted in different Asian countries, they used various methodologies and conceptualizations of advance care planning. We therefore aimed to summarize and systematically synthesize the evidence on native Asian patients’ perspectives on advance care planning and their underlying motives.

## Methods

This systematic review is reported according to the Preferred Reporting Items for Systematic Reviews and Meta-Analyses (PRISMA) 2020.^7^

### Design

This study obtained a phenomenological approach in which we integrated findings of primary quantitative and qualitative studies to build a network of related concepts that together provide a comprehensive understanding of Asian patients’ perspectives on advance care planning.^8–10^

### Data sources and searches

With the aid of a biomedical information specialist (WMB), we developed and deployed a systematic strategy for searching four electronic databases, EMBASE.com (1971-); MEDLINE ALL Ovid (1946-); Web of Science Core Collection (1975-); and Google Scholar from inception to July 27, 2020 (date last searched). Whenever applicable, search terms for each database were tailored using thesaurus terms (Emtree and MeSH; see Supplemental Appendix 1 for the full search strategies). The searches contained terms to describe advance care planning and advance directives, and were also designed to retrieve articles on end-of-life decision-making in Asian countries or among Asian populations. Conference papers, letters, notes, and editorials were excluded from the search, as were articles on children, and articles in languages other than English. We used no limit for publication date or study design. To ensure a comprehensive search, we scanned the reference lists in relevant literature reviews and in the included articles. Lastly, we inquired among different experts on advance care planning in Asia whether we had missed important studies that would met our inclusion and exclusion criteria.

### Study selection

Studies were included on the basis of the following inclusion criteria: an original empirical study published in English in peer-reviewed journals that focused on patients with serious illness living in the southern, eastern, and southeastern Asia; and that reported patients’ perspectives on advance care planning, their agreement or willingness to engage in it, the role of decision maker, and the motivational drivers for their willingness or unwillingness to engage in it.

We defined serious illness as a health condition that carried a high risk of mortality and either negatively impacted a person’s daily function or quality of life, or placed an excessive burden on their caregivers.^11^ This definition covers severe chronic conditions (such as cancer, renal failure, and advanced liver disease); dementia; and elderly patients living in long-term care facilities.

We further operationalized advance care planning as: (1) activities the authors had labeled as “advance care planning”; and/or (2) activities that involve patients, their family and/or healthcare professionals in discussions of the patients’ goals and/or preferences for future medical care and/or treatment; (3) activities that involve documentation processes of patients’ preferences, including (a) the appointment of a personal representative and (b) writing an advance directive.^2^ Due to the vast area of the Asian continent, we focused our search on its southern, eastern, and southeastern regions, whose cultural backgrounds are relatively comparable.^12^ We excluded studies on patients under 18 years old or on those diagnosed with mental disorders other than early dementia according to the criteria of Diagnostic and Statistical Manual of Mental Disorders V.^13^

On the basis of the inclusion and exclusion criteria, three authors (DM, MSK, and OG) were involved in independently screening titles and abstracts for eligibility and then reviewing the full-text articles. If necessary, disagreements were discussed and resolved with JR and/or CR. References were managed using Endnote bibliographic software version X9.

### Quality assessment and data extraction

Two of the three authors (DM and CPL or DM and OG) were involved in independently assessing the methodological quality of the included studies using the QualSyst tool, which has been described as suitable for various study designs.^14^ We employed the 10 standard criteria for qualitative studies and the 14 standard criteria for quantitative studies. Mixed-method studies were evaluated using both sets of criteria. We divided the sum of the scores by the total numbers of criteria. Any disagreements between reviewers were resolved through discussion. The summary scores were defined as strong (score of >0.80), good (0.71–0.80), adequate (0.51–0.70), or low (<0.50).^15^ Studies were not excluded on the basis of their methodological quality. To ensure that the quality assessment was free of bias, the author who conducted the quality assessment of an included study had not authored that specific paper.

A tailored data-extraction form was developed by DM. After piloting by JR, it was used by DM to extract data that included: (a) study characteristics; (b) patients’ perspectives on advance care planning, including their agreement with its concept and necessities, their willingness to engage in it, and their perspectives on the decision maker in it; (c) motives underlying patients’ willingness or unwillingness to engage in it. The extracted data was then reviewed by OG.

### Data synthesis and analysis

**Figure 1.**
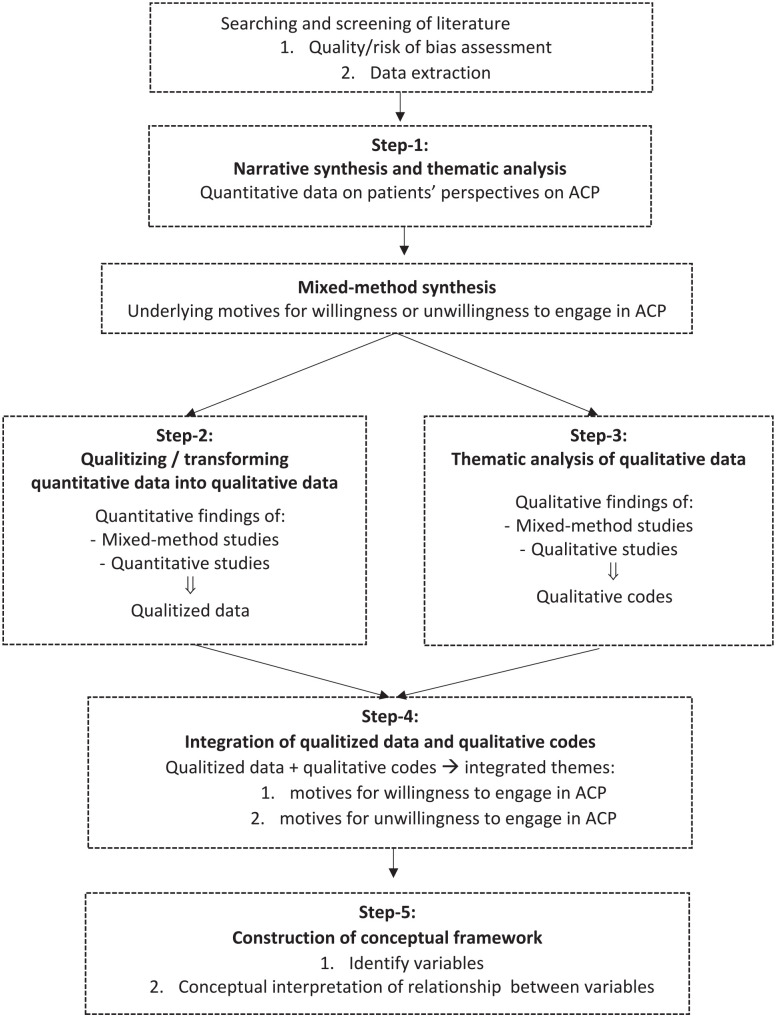
Multi-step synthesis and analysis. ACP: advance care planning.

Figure 1 shows the multi-step synthesis and analysis performed on the data. First, to explore patients’ perspectives on advance care planning, we conducted a narrative synthesis and thematic analysis according to Guidance on the Conduct of Narrative Synthesis in Systematic Reviews (Step-1),^16^ which includes textual description of the extracted data, tabulation, grouping, and clustering of data obtained from quantitative findings of quantitative or mixed-method studies. In the second step, we further synthesized patients’ underlying motives for willingness or unwillingness to engage in advance care planning, which we then analyzed on the basis of the type of data. The quantitative data was qualitized—that is, transformed into qualitative data—by attributing a qualitative thematic description to quantitative findings following the Bayesian conversion method.^17,18^ In the second step, the qualitative data was analyzed separately by DM and OG on the basis of Boeije’s^19^ procedure for thematic analysis. Any disagreements were resolved through consensus. In the fourth step, DM and OG further integrated the qualitized data with qualitative codes, using a data-based convergent integrative synthesis design to produce a set of integrated themes.^20^ This process was facilitated through a discussion with JR and CR. Qualitative analysis software (NVivo 12 Pro) was used to organize all qualitative data. Finally, in the fifth step we constructed a conceptual framework adapted from the Theory of Planned Behavior in order to visually display the interactions of the underlying motives with regard to patients’ willingness or unwillingness to engage in advance care planning.^9,21^

## Results

### Study characteristics

**Figure 2.**
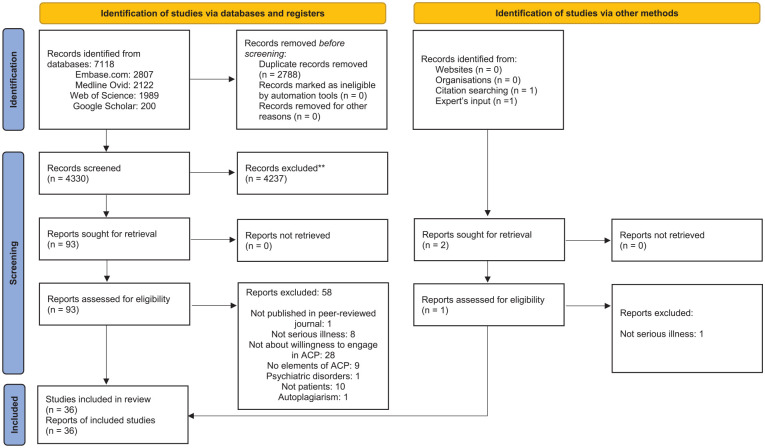
PRISMA flow diagram for study selection. ACP: advance care planning.

Through our systematic search, we identified 7118 potential studies. After de-duplication, 4330 studies remained, which were then screened on the basis of their titles and abstracts. We further excluded 4237 studies, primarily because they had not studied specific elements of advance care planning. After the addition of two studies identified by expert’s input and a manual search of reference lists, 94 studies were assessed full-text. Ultimately, 36 were included (Figure 2), 22 of which had used quantitative methods, 10 of which had used qualitative methods, and 4 of which had used mixed methods (Table 1 and Supplemental Appendix 2). A majority of the studies (*N* = 25) had been conducted in high-income countries^22^: Japan,^23–26^ South Korea,^24,27–35^ Hong Kong,^36–41^ Singapore,^42–44^ and Taiwan.^45–50^ The term advance care planning was used in 15 studies, most of which had been published in the last decade. Other studies, many of them less recent, used terms such as advance directive or do-not-resuscitate (DNR) order that were related mainly to advance care planning documents; or terms such as end-of-life discussion that were related to advance care planning. Fourteen studies conceptualized advance care planning as the completion of documents (advance directives or DNR orders), while 22 conceptualized advance care planning as a conversation process with or without documentation. Elderly patients (*n* = 16) and cancer patients (*n* = 14) were the most-studied patient populations. A majority of studies were conducted in a hospital-based setting (*n* = 23). Methodological quality was categorized as being strong in 11 studies, good in 11, adequate in 12, and low in 2 (Supplemental Appendices 3 and 4).

**Table 1.** Characteristics of the included studies (*n* = 36).

Study characteristics	*N* (%)
Type of study	
Quantitative study	22 (61)
Qualitative study	10 (28)
Mixed-methods study	4 (11)
Country/region^a^	
South Korea^a^	10
China^a^	6
Hong Kong	6
Taiwan	6
Japan^a^	4
Singapore	3
Malaysia	3
Term related to ACP used^b^	
Advance care planning	15
Term related to ACP documents:	
Advance directive	19
DNR order/directive	2
Physician order for life-sustaining-treatment	3
Term related to ACP conversation:	
End-of-life decision-making	5
Advance directive decision-making	1
The element of ACP studied	
ACP as completion of documents	14
ACP as process of discussion on preferences	13
Both	9
Number of patients in the study	
0–100	15
101–500	17
501–1000	1
>1000	3
Type of subjects studied	
Patients:	
- Cancer	14
- Non-cancer:	
Elderly with chronic serious illnesses	16
Chronic dialysis	1
- Not-specified non terminal serious illnesses	4
- Not-specified terminal illness	1
Setting	
Hospital	23
Palliative care unit or hospice	3
Elderly facility	9
No restriction in the setting	1

ACP: advance care planning; DNR: do-not-resuscitate.

a One study was conducted in South Korea, China, and Japan.

b Several studies used more than one terms related to advance care planning.

### Patients’ perspectives on advance care planning

#### Patients’ agreement with the importance of advance directive

Seven quantitative studies reported on whether or not patients thought advance directives were important^24,31,34,46,51,52,54^ (Supplemental Appendix 5). Three-quarters or more of Asian patients in six studies considered they were necessary: Malaysia (75%)^54^; South Korea (85%^24^; 87%^31^; 93%^34^); China (74%^51^; 80%^24^); Japan (96%)^24^; Taiwan (77%).^46^ In the seventh study, also from China, 22% of patients agreed on it.^51^

#### Patients’ willingness to engage in advance care planning or to draft an advance directive

Seven quantitative studies reported that 39%–90% of Asian patients were willing to engage in advance care planning (Table 2). Two of these reported that 62%–82% of patients’ were willing to engage in it together with their family or healthcare professionals. The first of these studies involved patients with advanced cancer in South Korea; 62% of these patients were willing to engage in advance care planning with their family, and 61% with healthcare professionals.^27^ In the second of these studies, from China, 82% of patients were willing to engage in advance care planning with their family and/or with their healthcare professionals).^55^ In Japan, the willingness to engage in advance care planning with the family (mean score 3.3 ± 0.61, range 1–4) was similar to the willingness to engage in advance care planning without families (mean score 3.2 ± 0.52) among older patients with chronic diseases.^23^ Four other studies reported Asian patients’ willingness to engage in advance care planning (39%–68%) without detailing their preferences on whom they would have the conversation with: Singapore (39%–49% of older patients with mild dementia)^43,44^; Taiwan (42% of nursing home residents)^49^; and Malaysia (68% of patients with kidney failure).^54^

**Table 2.** Patients’ willingness to engage in advance care planning or to draft an advance directive.

No	First author	Year	Country	Type of patient	Conceptualization of ACP	Patients’ willingness to engage in ACP	Percentage
1.	Cheong K^44^	2015	Singapore	Patients with early cognitive impairment	Advance care planning is a process that aims to inform and facilitate medical decision-making to reflect patients’ values and preferences in the event that they cannot communicate their wishes.	Willing to engage in ACP	39%
2.	Hing Wong A^54^	2016	Malaysia	Patients on routine hemodialysis	Advance care planning is a process of communication among the patients, their families, and professional caregiver, which include, but is not limited to discussing preferences for life-sustaining treatments.	Willing to engage in ACP	68%
3.	Lo TJ^43^	2017	Singapore	Patients with early cognitive impairment	Advance care planning is a process that facilitates decision-making on future care and helps patients with chronic or terminal illnesses make known their wishes before they lose their ability to do so.	Willing to engage in ACP	49%
4.	Sung HC^49^	2017	Taiwan	Elders living in long-term care facility	Advance care planning is a process of discussion between individuals and their physicians, formal caregivers, families, and friends about their preferences and wishes for future care if the individual lacks the capacity to express their wishes.	Willing to engage in ACP	42%
5.	Hou XT^55^	2018	China	Patients with advanced cancer	Advanced care planning is the process whereby there is a discussion between individuals and their physicians, family, and friends about their preferences and wishes for future care at a time when they may lack the capacity to express such wishes.	Willing to engage in ACP: (a) With HCPs and families (b) With HCPs only (c) With families only	a) 59% b) 12% c) 11%
6.	Kizawa Y^23^	2020	Japan	Elderly patients with chronic disease	Not defined	Willingness to engage in ACP^a^: (a) By themselves (b) With families (Mean score ± SD; Range: 1–4)	a) 3.2 ± 0.52 b) 3.3± 0.61
7.	Yoo SH^27^	2020	South Korea	Patients with advanced solid and/or hematologic cancer	Not defined	(a) Willing to engage in ACP with family • In total • Among those who understand their illness • Among those who don’t understand their illness (b) Willing to engage in ACP with physician • In total • Among those who understand their illness • Among those who don’t understand their illness	(a) 62% • 67% • 58% (b) 61% • 68% • 56%
No	First author	Year	Country	Type of patient	Conceptualization of AD	Patients’ willingness to draft an AD	Percentage
1.	Chu LW^39^	2011	Hong Kong	Elderly living in long-term care facility	An advance directive is a statement, usually in writing, in which a person, when mentally competent, indicates the form of healthcare he or she would like to have in a future time when he or she is no longer competent	Willing to draft an AD	88%
2.	Ting FH^37^	2011	Hong Kong	Elderly in-patients with chronic diseases	Not defined	Willing to draft an AD if formally legalized	49%
3.	Ni P^56^	2014	China	Elders living in long-term care facility	An advance directive is a legal document that outlines a person’s care preferences and wishes, should their decision-making ability be diminished as a result of a critical illness or cognitive impairment.	Willing to draft an AD	32%
4.	Park J^35^	2016	South Korea	Elders living in long-term care facility	An advance directive is a written document specifying medical treatments that people want or do not want to receive in the event where the ability to communicate or make decisions is lost due to a progression of illness.	Willing to draft an AD	59%
5.	Hui EC^38^	2017	Hong Kong	Patients with solid cancer (any stage)	Not defined	Willing to draft an AD	22% (and list treatment preferences); 12% (and assign proxy decision-maker)
6.	An HJ^30^	2019	South Korea	Patients with terminal cancer	An advance directive is a legal document written by anyone regardless of his/her illness, and it includes a future medical care plan, living will, or designation of power of attorney. The POLST form is a medical document that mainly pertains to a patient’s future care, including end of life care preferences in case they lose the capacity to make decisions.	Willing to sign AD (POLST)	52%
7.	Kim JW^28^	2019	South Korea	Patients with advanced solid cancer	POLST is a part of an advance care planning with advance directives and is written by a doctor based on the patient’s wishes at the terminal stage.	Willing to draft AD (POLST)	71%
8.	Park HY^33^	2019	South Korea	Patients with cancer (any stage)	Advance directives are statement that an adult could write about the determination of life-sustaining treatment and utilization of hospice at a terminal stage.	Willing to draft an AD: (a) In a healthy condition (b) When diagnosed with serious illness (c) When the terminal stage is difficult to predict (d) When the condition of serious illness worsened (e) When the terminal stage is easy to predict (f) When diagnosed with terminal stage	(a) 59% (b) 69% (c) 68% (d) 73% (e) 73% (f) 74%
9.	Feng C^53^	2020	China	Patients with lung cancer (any stage)	Advance directives are legal documents in which people choose the medical treatments they are, or are not, willing to receive if in the future they lose the capacity to talk about their wishes.	Willing to sign AD	80%
10.	Yoo SH^27^	2020	South Korea	Patients with advanced solid and/or hematologic cancer	Not defined	(a) Willing to draft an AD: • Among those who understand their illness • Among those who don’t understand their illness (b) Willing to draft POLST • Among those who understand their illness • Among those who don’t understand their illness	(a) • 55% • 45% (b) • 59% • 49%

ACP: advance care planning; AD: advance directive, CPR: cardiopulmonary resuscitation; HCPs: healthcare professionals; POLST: physician order for life-sustaining-treatment; SD: standard deviation.

a Higher score indicates greater willingness.

Ten studies reported that 32%–88% of Asian patients were willing to draft an advance directive: Hong Kong (88% of nursing home residents, 49% of critically-ill elderly patients, and 34% of cancer patients)^37–39^; China (32% of nursing home residents and 80% of cancer patients)^53,56^; and South Korea (52%–74% of advanced cancer patients; 59% of nursing home residents).^27,28,30,33,35^

#### Patient’s perspectives on the decision maker in advance care planning

Seven quantitative studies reported the perspectives of Asian patients on their own role, and the roles of their family and physicians, regarding decision-making in advance care planning (Supplemental Appendix 6). Fifty-one to ninety-five percent of Asian patients considered the main decision maker in advance care planning to be themselves, either alone or together with their family members and/or physicians.^24,26,28,31,37,38^ Five to thirty-one percent of Asian patients preferred their family or physician to be the main decision maker in advance care planning.^24,26,28,31,37,38^

Four studies compared preferred styles of decision-making, reporting a stronger preference for collective decision-making (i.e. patients together with their family and/or their physicians) than for individualistic decision-making: Japan (61% vs 33%),^24^ South Korea (67% vs 27%),^24^ China (48% vs 26%),^24^ and Hong Kong (71% vs 21%).^38^ These findings contrast with two studies among older people with serious illnesses in which individualistic decision-making was preferred: in Hong Kong (14% vs 55%)^37^ and South Korea (32% vs 39%).^31^

### Underlying motives for patients’ willingness or unwillingness to engage in advance care planning

Twenty-two studies (8 quantitative, 10 qualitative, and 4 mixed-method) examined patients’ underlying motives for being willing or unwilling to engage in advance care planning. We summarized the quantitative data in Supplemental Appendix 7 and further transformed them into qualitized data (Table 3). Our analysis of the qualitative data produced 29 qualitative codes (Supplemental Appendix 8), 5 related to willingness, and 24 related to unwillingness to participate in advance care planning.

**Table 3.** Underlying motives for patients’ willingness or unwillingness to engage in advance care planning.

Motivational drivers for engagement in advance care planning			
Qualitized data	Qualitative codes	Integrated themes	Conceptual framework variables
Patients’ belief that ACP would ensure their wishes to be respected^37^	Patients’ belief that ACP would promote autonomy^25,42,44,57,58^	Patients’ belief that ACP would promote autonomy	Behavioral beliefs
Patients’ awareness of future incapacity^35,56^			
Patients’ wish to exercise self-determination^28,35^			
Patients’ belief that ACP would ensure a comfortable end of life^37^	Patients’ wish to have comfort near the end of their life^40,57,58^	Patients’ belief that ACP would enable a comfortable end of life	
Patients’ belief that quality of life is more important than length of life^37^			
Patients’ belief that ACP would prevent them from the suffering due to meaningless treatment^28^			
Patients’ belief that ACP would avoid causing burden to the family with end of life decision^35,37^	Patients’ wish to avoid being a burden to their family^25,42,44,57^ or the society^47^	Patients’ belief that ACP would avoid causing burden to the family or society	
Patients’ belief that ACP would avoid burdening the society^37^			
Patients’ wish to ease the economic burden on the family^28^			
Patients’ belief that ACP would prevent conflict between family members^37^	Patients’ belief that ACP would create connection with the family^42^	Patients’ belief that ACP would facilitate shared understanding between patient and family	
Patients wish that ACP would help family understand their wishes at an early stage^56^			
Patients’ experience with the death of a relative/friend^37^	Patients’ positive experience with ACP^45,58^	Patients’ belief that ACP is beneficial after their experience with end of life or ACP	
Patients’ religious beliefs^37^		Patients’ religious beliefs	Normative beliefs
Patients’ wish to follow physician’s recommendation for ACP^28^		Patients’ wish to follow physician’s recommendation for ACP	
Motivational drivers for non-engagement in advance care planning			
Qualitized data	Qualitative codes	Integrated themes	Conceptual framework variables
Patients’ lack of knowledge of own disease state^30^	Patients’ lack of illness understanding^25,36,44,57^	Patients’ lack of illness understanding	Knowledge
Patients’ concern of lacking the information needed for decision-making^55^			
Patients’ lack of awareness of AD^35,37,56^	Incomplete understanding/lack of awareness regarding ACP^41–44,48,50,57,58^	Patients’ limited understanding of ACP	
Patients’ lack of knowledge about AD^30,33^	Patients’ lack of understanding of ACP relevance for planning beyond financial arrangements^43,44^		
Patients’ need of more information^38^			
Patients’ lack of understanding of the policy^28^			
Patients’ lack of idea on how to approach end of life communication^55^			
Patients’ belief that ACP is not useful^56^	Patients inability to appreciate what intent of ACP^43,50^	Patients’ belief that ACP is not necessary or beneficial	Behavioral beliefs
Patients’ belief that talking about ACP would make their relatives sad^55^	Patients’ concern that ACP would cause distress or burden for family members^41,42,48,50,58^	Patients’ concern of implications of ACP	
	Patients’ concern that ACP would cause conflict within their family members^44,50,58^		
Patients’ concern of the psychological discomfort produced when thinking about a terminal illness^33^	Patient’s concern that they would feel uncomfortable discussing end of life issues/lose of hope^29,41,42^		
Patients’ discomfort in talking about death^30^			
Patients’ belief that talking about ACP would make them sad^55^			
Patients’ belief that drafting AD would mean giving up or result to being abandoned by the physicians^30^	Patient’s belief that discussing end of life would bring bad luck (taboo)^50,58^		
Patients’ belief that signing AD would lead to bad things^30^			
Patients’ uncertainty whether their wish would be respected^33^	Patients’ doubted about the effectiveness of ACP in conveying their wishes^44^	Patients’ doubted about the effectiveness of ACP in conveying their wishes	
Motivational drivers for non-engagement in advance care planning			
Qualitized data	Qualitative codes	Integrated themes	Conceptual framework variables
	Patients’ belief that family does not support their engagement in ACP^43,44,47^	Patients’ belief that family does not support their engagement in ACP	Normative beliefs
	Patients’ belief that HCPs do not advocate ACP^41,43^	Patients’ belief that HCPs do not advocate ACP	
Patients’ wish to let the nature take its course^37^	Patients’ wish to seek harmony with the mandate of nature^50^	Patients’ belief that ACP goes against their faith/religious beliefs	
Patients’ religious beliefs^37^	Patients’ belief in providence^41,44,48,50,57,58^		
Patients’ concern of difficulties of making decisions in advance^38^	Patients’ concern of difficulty in planning for the unknown/unpredictable disease course^25,41,45,50^	Patients’ concern of difficulty in planning for the unknown	Control beliefs
Patients’ concern that their decision may change later^33,37^	Patients’ concern that their decisions may change in the future^29,42^		
	Patients considered ACP irrelevant due to their socioeconomic dependency^25,43,44,58^	Patients’ sense of limited options for future care	
	Patients’ belief of limited options available for them in the future care^25^		
	Patients’ belief that limited care continuity hampers ACP^41^	Patients’ sense of the lack of healthcare supporting system for ACP	
	Patients’ belief that time constraint from HCPs side hampers ACP^41^		
	Patients’ belief that HCPs lack the communication skills and empathy for ACP^41^	Patients’ belief that HCPs lack the skills for ACP	
Willingness to engage in ACP in particular approaches			
Qualitized data	Qualitative codes	Integrated themes	Conceptual framework variables
Patient act as sole primary decision maker in ACP^24,28,31,37,38^	Patient as independent decision maker in ACP^25,42,57,58^	Patients’ preference for active involvement in decision-making, individually	Actors and roles
Patient, together with family and/or HCPs, as decision maker in ACP^24,31,37,38^	Patient, together with family and/or HCPs, as decision maker in ACP^42^	Patient preference for active involvement in decision-making, together with the family and/or HCPs	
Patients’ wish to discuss with the family^28^			
Patients’ wish to entrust decision-making to the relatives^30,35,37,38,55,56^	Patients’ wish to entrust decision-making to family members^25,36,41–44,50,57,58^	Patients’ preference for passive involvement in decision-making	
Patients belief the family will make the best decision on their behalf^33,43^			
Patients’ wish to entrust decision-making to the physicians^30,35,37,55^	Patients’ belief that the physicians would “do what is right”^41,50,57,58^		
Patients’ belief that there is no need to think about drafting an AD now^37^	Patients’ belief that it’s too early to engage in ACP^25,50^	Patients’ preference of timing for initiation of ACP	Timing
Patients’ belief that it’s too early for ACP^56^			
Patients’ belief that ACP is not necessary in their current age^35^			
Patients’ belief that it’s not the right time yet^28^			
Patients’ need of more time to think^28,38^			
Patients belief that drafting an AD is important^24,31,34,54^		Patients’ preference of ACP formality	Formality
Patients’ preference to further discuss with family^43^	Patients’ belief that informal planning would suffice^29,44,57^		

ACP: advance care planning; AD: advance directive, HCPs: healthcare professionals.

By integrating the qualitized and qualitative data, we developed seven integrated themes regarding patients’ motives for willingness to engage in advance care planning (Table 3): (a) their belief that it would promote autonomy; (b) their belief that it would enable a comfortable end-of-life; (c) their belief that it would avoid burden on the family; (d) their belief that it would facilitate shared understanding between patient and family; (e) their past experiences with end-of-life or advance care planning; (f) their religious beliefs; and (g) their wish to follow their physician’s recommendations.

Eleven integrated themes were developed as motives for patients’ unwillingness to engage in advance care planning: (a) their lack of understanding of their illness; (b) their limited understanding of advance care planning; (c) their concerns about its implications; (d) their belief that it was not necessary or beneficial; (e) their uncertainty about its effectiveness in conveying their wishes; (f) their belief that healthcare professionals did not advocate advance care planning; (g) their belief that family did not support their engagement in it; (h) their belief that it went against their faith or religious beliefs; (i) their sense that the options for future care were limited; (j) their sense that it was not yet partially or fully supported by the healthcare system; and (k) their belief that healthcare professionals lacked the skills needed for advance care planning.

### Conceptual framework for patients’ willingness to engage in advance care planning

**Figure 3.**
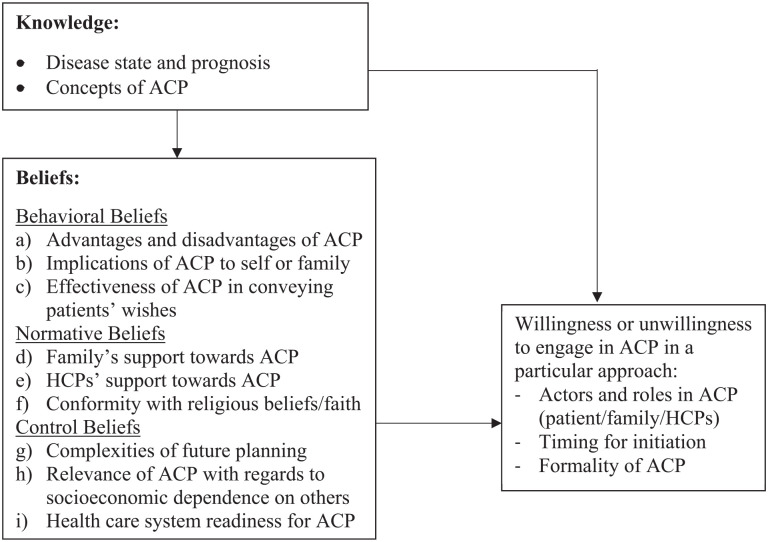
Conceptual framework for patients’ willingness to engage in ACP. ACP: advance care planning; HCPs: healthcare professionals.

Next, we used these integrated themes to develop a conceptual framework organized on the basis of knowledge, beliefs, and willingness to engage in advance care planning (Figure 3). According to the Theory of Planned Behavior,^21^ beliefs in advance care planning were further divided into three types: (a) behavioral beliefs in advance care planning (i.e. patients’ beliefs regarding the likely consequences of engaging in advance care planning); (b) normative beliefs in advance care planning (i.e. the normative expectations of others regarding their engagement in advance care planning); and (c) control beliefs in advance care planning (i.e. the presence of factors that might facilitate or hinder their engagement in advance care planning).

#### Patients’ knowledge

Patients who lacked awareness of their disease severity and prognosis^25,30,36,44,55,57,58^ and/or knowledge regarding advance care planning^27,28,30,33,35,37,38,41–44,48,50,55–58^ were less likely to engage in it. For instance, patients who had mistakenly understood that advance care planning was merely a discussion about financial arrangements decided not to engage in it if their planning was already sufficient or if they had no assets to plan for.^43,44^ Our model was based on the hypothesis that patients’ beliefs and willingness to engage in advance care planning were influenced by their knowledge of its concept and of their illness.

#### Patients’ behavioral beliefs about advance care planning

Studies reported that patients’ beliefs about the benefits of advance care planning were important motivators of their engagement in it; such benefits include the belief that advance care planning promoted autonomy,^25,28,35,37,42,44,56–58^ enabled a comfortable end-of-life,^28,37,40,57,58^ avoided burdening family members,^25,28,35,37,44,57^ and facilitated shared understanding with family members.^37,42,56^ Conversely, five groups of patients would be less likely to engage in advance care planning: (a) those who believed that it was not beneficial ^43,56^; (b) those who believed that engaging in it might cause conflict between their family members or distress to them^41,42,44,48,50,55,58^ or to themselves^29,30,33,41,42,55^; (c) those who believed that discussing death would bring bad luck^30,50,58^; (d) those who believed that signing the advance care planning document would lead to substandard care^30^; and (e) those who were not sure that it would guarantee their wishes were respected.^33,44^

#### Patients’ normative beliefs about advance care planning

We identified three normative components of beliefs pertaining to engagement in advance care planning. The first was related to family: patients who believed that their family did not support their engagement in advance care planning^43,44,47^ would be less likely to engage in it. The second was related to healthcare professionals: patients would be less likely to engage in advance care planning if their physicians did not advise them to do so.^41,43^ The third was related to faith or religious belief. Seven studies found that patients’ faith or spiritual beliefs were motives for non-engagement in advance care planning.^37,41,44,48,50,57,58^ Like those who believed that their future was predetermined by God or their past actions and those who believed in the mandate of nature would be likely to accept what they regarded as their predetermined fate rather than attempting to take control of it or modify it through advance care planning.

#### Patients’ control beliefs about advance care planning

Patients were particularly concerned about the complexities of advance care planning with regard to the difficulties of planning for the unknown^25,38,41,45,50^ and the possibility of a future change of mind.^29,33,37,42^ As their socioeconomic dependency on others gave them only limited options for future care, they were concerned that planning for various future scenarios might not be relevant to them.^25,44,58^ Patients were also concerned that, as they had never had the chance to develop a long-term relationship with a healthcare professional that would make advance care planning possible, the healthcare system might not be supportive of it.^41^ They were also concerned that healthcare professionals lacked the skills and empathy needed to engage in it.^41^

#### Patients’ willingness or unwillingness to engage in advance care planning

Our data also shows that willingness or unwillingness depended on three factors: (a) which role people have in advance care planning; (b) when it is initiated; and (c) how formally it is carried out. Patients tended to expect one of the following: (a) active engagement that involved the patient with their family members and/or healthcare professionals^24,28,31,37,38,42^; or (b) passive involvement in which they preferred to extend their autonomy and entrust decision-making to their family members or healthcare professionals.^25,30,33,35–38,41–44,50,55–58^ The motivations for entrusting decision-making to family included beliefs that: the family knew the appropriate decision for the patient,^41,43,44,50^ such decision making was the children’s responsibility to the parents,^50^ family would carry out the patient’s wishes,^43^ and the patients would have no control over future decision-making.^58^ A further motivation was patients’ experience of being treated well by the family.^25^ A reason for entrusting decision-making to physicians was a belief that physicians would do what was best for the patient.^41,50,57,58^ Those who preferred to be their own primary decision maker were motivated by their doubts that the family would honor their wishes,^57^ and by their expectation that they would be able to maintain control of their life.^25,58^

Our findings also show that patients were willing to initiate advance care planning at a particular time in the future or later in the course of their illness.^25,28,33,35,37,38,50,56^ With regard to patients’ preferences for documenting their conversations, our findings were varied: while some preferred a written document,^24,31,34,54^ others preferred verbal communication with their family, and/or healthcare professionals without drafting or signing a written document.^29,43,44,57^

## Discussion

To better understand Asian patients’ perspectives on advance care planning and the motives underlying their willingness or unwillingness to engage in it, we systematically synthesized and integrated outcomes from different types of studies, and then developed a conceptual framework on the basis of our findings. Most of these findings originated in high-income Asian countries. Acknowledging the limit we set to our search, the term “Asian patients” we used to describe our findings refers to Asian patients in southern, southeastern, and eastern Asia. Our most important finding is that a majority of Asian patients agreed that advance care planning was necessary. The main motive for their engagement in it concerned its benefits, such as promoting autonomy, allowing a comfortable end of life, avoiding burden on family members, and facilitating shared understanding with family members. Conversely, a range of motives characterized those who were unwilling to engage in it: patients’ lack of understanding of their disease, their misperceptions about advance care planning, and the following beliefs: that it was not beneficial, that it was potentially harmful, that it was not consistent with their religious beliefs or with the wishes of their family or healthcare professionals, and that there were various barriers to it. Our findings suggest that Asian patients would benefit from an individual approach with regard to the individual(s) who should communicate values or be present during advance care planning, the right time for initiating advance care planning conversations, and the formality of advance care planning.

Our study confirms previous findings suggesting that proper understanding of their illness (e.g. prognosis) is an important initial step to patients’ realization of whether or not they would need further conversations on their goals and future care plan.^59,60^ The poor illness understanding identified in our study is likely to have been caused by limited truth-telling—a common aspect of communication with seriously ill patients in Asia,^6^ which leads to their exclusion from conversations about poor diagnosis and prognosis. Healthcare professionals’ tendency toward partial disclosure or non-disclosure is not compatible with most Asian patients’ reported preference for truth-telling communication.^61–64^ Our study thus provides further confirmation of the fact that clarifying patients’ understanding of their illness (including prognosis) by encouraging truth-telling communication is an important prerequisite for engagement in advance care planning.

Our study also shows that Asian patients have only a limited understanding of what advance care planning entails. Three misperceptions of advance care planning are particularly common: that it is purely a financial planning process, a completion of a formal document, or a conversation related to death and dying. These may be due to the facts that advance care planning is a relatively new concept in Asia that is both complex and continuously evolving, various terms of legislation on advance directives in different countries, and that there is little or no public education on it in Asia.^3,65^ Correcting these misperceptions whilst simultaneously taking proper account of the Asian context—for example by engaging family members earlier—is central to the promotion of positive attitudes to it. A similar phenomenon has been reported by studies from non-Asian countries, which solidify the influence of participants’ knowledge regarding advance care planning on its delivery across different cultures.^66–68^

Our earlier systematic review showed that Asian healthcare professionals rarely engaged patients in advance care planning and, in the event of disagreement between patients’ advance directive and the family’s wishes, would defer to the family.^6^ However, it is clear from our current findings that a meaningful number of Asian patients expect and prefer active participation in advance care planning, either together with their families, or, to a lesser extent, individually. This suggests that the commonly stereotyped Asian values of passive or family-centered decision-making may in fact be too narrow, and, due possibly to modernization and globalization, that a shift may also be taking place toward more autonomous forms of decision-making.^69^ This evidence further emphasizes the importance of avoiding East-West cultural stereotypes and of identifying individual patients’ personal values and preferences for engaging in medical decision-making.

Other important motives for patients’ willingness or unwillingness to engage in advance care planning are beliefs about its harms and benefits. Central to these beliefs is the motivation to protect oneself and one’s loved ones from future suffering, whether (a) physical (such as that due to unwanted treatment in the absence of advance care planning, or to substandard treatment after signing an advance directive); (b) financial (such as that caused by economic burdens on the family); (c) social (such as that due to family conflict); or (d) psychological (such as the distress caused by decision-making as a surrogate or by loss of hope).

Our findings also suggest that certain normative beliefs play an important role in patients’ engagement in advance care planning. Asian patients will favor advance care planning when it is in accordance with a physician’s advice, families’ wishes, or patients’ religious beliefs about the end of life. Particularly in Asian collectivist culture, it is essential to seek harmony with others, including family members, society, and nature. While death is often regarded as God’s will or the mandate of nature, discussing it openly may also be believed to cause bad luck. Open and honest communication on these beliefs and related concerns is therefore essential, not only to allow misperceptions or false beliefs to be corrected, but also to allow approaches to the topic that are more acceptable to a specific patient’s personal values. Acknowledging such beliefs is essential to facilitating an appropriate and patient-centered approach to advance care planning.

Our model also suggests that these beliefs have led to various preferences for role in advance care planning, one of which involves granting autonomy to their family or healthcare professionals, and thus allowing their own values to be communicated, and decisions to be made, by family or healthcare professionals. In this case, advance care planning should facilitate mutual understanding of patients’ values. This would allow for the further translation of these values into relevant goals and preferences without limiting the context of conversations and the patient’s eventual role in the process.

## Strengths and limitations

To the best of our knowledge, this is the first systematic review to explore Asian patients’ perspectives on and willingness to engage in advance care planning, and also their underlying motives for this. As advance care planning is an emerging concept in Asia, our comprehensive conceptualization of it made it possible to conduct a sensitive search that did not necessarily use advance care planning as a search term, but nonetheless identified studies examining its relevant elements. The use of mixed-method systematic review enabled us to gain a deeper understanding of the findings by integrating different types of evidence from various types of studies.

When interpreting this systematic review, three main limitations should be taken into account. Firstly, our inclusion solely of studies published in English may have led valuable contributions to be excluded. However, we believe that our comprehensive search strategy, wide inclusion criteria, and mixed-method strategy enabled us to identify sufficient number of studies to answer our research questions. Secondly, there was a possibility of selection bias, as patients with a greater interest in advance care planning may have been more inclined to participate in the studies in question. Finally, our results may lack generalizability to low and middle-income Asian countries, other regions of Asia (i.e. northern, western, and central Asia), and patients with mental disorders.

## What this review adds

Our study suggests the importance of developing a culturally sensitive model of advance care planning for Asia. Because decision-making in Asia is primarily family driven, advance care planning should focus on achieving a shared understanding of patients’ values by encouraging open communications and establishing the connection between patients and their family. Our findings may also be relevant to the practice of advance care planning in Western countries, particularly when engaging patients or family members of Asian descent. Healthcare professionals who engage in advance care planning with patients of Asian origin should avoid stereotyping Asian collectivist culture and bear in mind that these patients may prefer active involvement in it. To facilitate a proper approach to advance care planning conversations, healthcare professionals should also familiarize themselves with various beliefs about advance care planning that are commonly found in Asian culture. With regard to these beliefs, our findings suggest that the focus of advance care planning conversations should be shifted from merely communicating care objectives toward exploring and establishing values, and thereby achieving truly value-concordant care. A separate review is currently underway and aims to explore whether the phenomenon in Asians living in foreign countries is comparable to our current findings and how acculturation may play role in it.^70^

## Conclusion

The essential first steps toward engaging Asian patients in advance care planning involve a process of education and clarification, in which various misperceptions about their illness and prognosis are resolved, and it is clearly established what advance care planning entails. Advance care planning for Asian patients should be able to accommodate the diversity of patients’ beliefs; their preferences with regard to their role in it, either as active participants, or by delegating responsibility to family members or healthcare professionals; decisions on the best time to initiate it; and decisions on formally documenting it.

## Supplemental Material

sj-pdf-1-pmj-10.1177_02692163211042530 – Supplemental material for Asian patients’ perspectives on advance care planning: A mixed-method systematic review and conceptual framework Click here for additional data file. Supplemental material, sj-pdf-1-pmj-10.1177_02692163211042530 for Asian patients’ perspectives on advance care planning: A mixed-method systematic review and conceptual framework by Diah Martina, Olaf P Geerse, Cheng-Pei Lin, Martina S Kristanti, Wichor M Bramer, Masanori Mori, Ida J Korfage, Agnes van der Heide, Judith AC Rietjens and Carin CD van der Rijt in Palliative Medicine
