# Supplementary material for: Asian patients’ perspectives on advance care planning: A mixed-method systematic review and conceptual framework
Source: Palliat Med. 2021 Sep 6;35(10):1776–92. doi: 10.1177/02692163211042530 (PMC8637390; doi:10.1177/02692163211042530)
Supplement: sj-pdf-1-pmj-10.1177_02692163211042530 – Supplemental material for Asian patients’ perspectives on advance care planning: A mixed-method systematic review and conceptual framework [file sj-pdf-1-pmj-10.1177_02692163211042530.pdf]

Appendices

Appendix 1. Search strategies

Medline Ovid

(exp Advance Directives/ OR Resuscitation Orders/ OR ((Decision Making/ OR Communication/ OR Physician-Patient Relations/ OR Patient Preference/ OR Personal Autonomy/ OR Knowledge/) AND (Terminal Care/ OR Palliative Care/ OR Terminally Ill/OR Resuscitation/ OR Life Support Care/ OR Euthanasia/ OR Hospice/)) OR (((Advance) ADJ3 (plan* OR directive*)) OR ((living-will*)) OR ((decision* OR decid* OR plan OR plans OR planning OR preference* OR want OR wish* OR dilemma* OR refus* OR choos* OR choice* OR communication OR talking OR disclos* OR autonom* OR attitude* OR practice* OR perspective*) ADJ6 (terminal* OR end of life OR palliativ* OR serious*-ill* OR severe*-ill* OR death OR dying OR advanced*-cancer* OR euthanas* OR hospice*)) OR ((do-not OR refus*) ADJ3 resuscit*) OR ((decision* OR decid* OR plan OR plans OR planning OR preference* OR want OR wish* OR dilemma* OR refus* OR choos* OR choice* OR communication OR talking OR disclos* OR autonom* OR attitude* OR practice* OR perspective*) ADJ6 life ADJ (saving OR saver* OR sustain* OR resuscit* OR threat* OR support*))).ab,ti.) AND (Asia/ OR exp Asia, Southeastern/ OR exp Far East/ OR Asia, Western/ OR Bangladesh/ OR Bhutan/ OR exp India/ OR Nepal/ OR Pakistan/ OR Sri Lanka/ OR Asian Continental Ancestry Group/ OR (Asia* OR Afghan* OR Bangla* OR Bhutan* OR Borne* OR Brunei* OR Cambod* OR China* OR Chinese* OR India OR Indonesia* OR Japan* OR Korea* OR Laos* OR Laotion* OR Malaysia* OR Mongolia* OR Myanmar* OR Birmese* OR Birma OR Nepal* OR Pakistan* OR Papua* OR Philippin* OR Singapore* OR Sri-Lank* OR Taiwan* OR Thailand* OR Thai OR Timor* OR Viet-Nam* OR VietNam* OR mekong OR (eastern NOT ((middle OR mediterr* OR europe) ADJ3 eastern)) OR far-east).ab,ti,jn,cp.) NOT (exp Emigration and Immigration/ OR exp Tissue Donors/ OR exp Transients and Migrants/ OR exp transplantation/ OR (immigr* OR migrant* OR emigra* OR refugee* OR donor* OR donation OR transplant* OR chinese american* OR japanese american* OR korean american* OR asian american*).ab,ti.) NOT (letter* OR news OR comment* OR editorial* OR congres* OR abstract* OR book* OR chapter* OR dissertation abstract*).pt. AND english.la. NOT (exp child/ NOT exp adult/

Web of science

TS=((((("Advance") NEAR/2 (plan* OR directive*)) OR ((living-will*)) OR ((decision* OR decid* OR "plan" OR "plans" OR "planning" OR preference* OR "want" OR wish* OR dilemma* OR refus* OR choos* OR choice* OR "communication" OR "talking" OR disclos* OR autonom* OR attitude* OR pratice* OR perspective*) NEAR/5 (terminal* OR "end of life" OR palliativ* OR serious*-ill* OR severe*-ill* OR death OR dying OR advanced*-cancer* OR euthanas* OR hospice*)) OR (("do-not" OR refus*) NEAR/2 resuscit*) OR ((decision* OR decid* OR "plan" OR "plans" OR "planning" OR preference* OR "want" OR wish* OR dilemma* OR refus* OR choos* OR choice* OR "communication" OR "talking" OR disclos* OR autonom* OR attitude* OR pratice* OR perspective*) NEAR/5 life NEAR/1 (saving OR saver* OR sustain* OR resuscit* OR threat* OR support*)))) AND ((Asia* OR Afghan* OR Bangla* OR Bhutan* OR Borne* OR Brunei* OR Cambod* OR China* OR Chinese* OR India OR Indonesia* OR Japan* OR Korea* OR Laos* OR Laotion* OR Malaysia* OR Mongolia* OR Myanmar* OR Birmese* OR Birma OR Nepal* OR Pakistan* OR Papua* OR Philippin* OR Singapore* OR Sri-Lank* OR Taiwan* OR Thailand* OR Thai OR Timor* OR Viet-Nam* OR VietNam* OR mekong OR ("eastern" NOT (("middle" OR mediterr* OR "europe") NEAR/2 "eastern")) OR far-east)) NOT ((immigr* OR migrant* OR emigra* OR refugee* OR donor* OR donation OR transplant* OR "chinese american*" OR "japanese american*" OR "korean american*" OR "asian american*")) NOT (child* NOT adult*))

Embase.com

('living will'/exp OR (('patient decision making'/exp OR 'decision making'/de OR 'interpersonal communication'/exp OR 'doctor patient relation'/de OR 'patient information'/de OR 'patient preference'/de OR 'patient autonomy'/de OR 'personal autonomy'/de OR 'patient attitude'/de OR 'knowledge'/exp OR 'personal experience'/de) AND ('terminal care'/exp OR 'palliative therapy'/exp OR 'terminally ill patient'/exp OR 'terminal disease'/de OR 'life threat'/exp OR 'advanced cancer'/de OR resuscitation/de OR 'life sustaining treatment'/de OR euthanasia/de OR hospice/de)) OR (((Advance) NEAR/3 (plan* OR directive*)) OR ((living-will*)) OR ((decision* OR decid* OR plan OR plans OR planning OR preference* OR want OR wish* OR dilemma* OR refus* OR choos* OR choice* OR communication OR talking OR disclos* OR autonom* OR attitude* OR pratice* OR perspective*) NEAR/6 (terminal* OR 'end of life' OR palliativ* OR serious*-ill* OR severe*-ill* OR death OR dying OR advanced*-cancer* OR euthanas* OR hospice*)) OR ((do-not OR refus*) NEAR/3 resuscit*) OR ((decision* OR decid* OR plan OR plans OR planning OR preference* OR want OR wish* OR dilemma* OR refus* OR choos* OR choice* OR communication OR talking OR disclos* OR autonom* OR attitude* OR pratice* OR perspective*) NEAR/6 life NEXT/1 (saving OR saver* OR sustain* OR resuscit* OR threat* OR support*))):ab,ti) AND ('Asia'/de OR 'Asian'/de OR 'South Asian'/exp OR 'Southeast Asian'/exp OR 'Far East'/exp OR 'South Asia'/exp OR 'Japanese (people)'/exp OR 'Korean (people)'/exp OR 'Sino-Tibetan people'/exp OR (Asia* OR Afghan* OR Bangla* OR Bhutan* OR Borne* OR Brunei* OR Cambod* OR China* OR Chinese* OR India OR Indonesia* OR Japan* OR Korea* OR Laos* OR Laotion* OR Malaysia* OR Mongolia* OR Myanmar* OR Birmese* OR Birma OR Nepal* OR Pakistan* OR Papua* OR Philippin* OR Singapore* OR Sri-Lank* OR Taiwan* OR Thailand* OR Thai OR Timor* OR Viet-Nam* OR VietNam* OR mekong OR (eastern NOT ((middle OR mediterr* OR europe) NEAR/3 eastern)) OR far-east):ab,ti,ta,cy) NOT ('immigration'/exp OR 'donor'/exp OR 'migrant'/exp OR 'transplantation'/exp OR (immigr* OR migrant* OR emigra* OR refugee* OR donor* OR donation OR transplant* OR 'chinese american*' OR 'japanese american*' OR 'korean american*' OR 'asian american*'):ab,ti) NOT ([Conference Abstract]/lim OR [Letter]/lim OR [Note]/lim OR [Editorial]/lim) AND [english]/lim NOT (child/exp NOT adult/exp)

Google scholar (top 200 ranked)

"living will|wills"|"advance directive|directives"|"advance care planning|plans|plan" Asia|China|Chinese|India|Indonesia|Japan|Japanese|Korea -immigration -donor -migrant -transplantation -american

Appendix 2. Description of included studies

No.	First author (year)	Study Design	Country or region	Setting	Subjects	Number of participants	Element of ACP being studied	Term related to ACP studied
1.	Voltz R (1998)[26(a)]	Cross sectional survey	Japan	Hospice or palliative care unit in hospital	Patients with terminal illness	34 (out of 252). Other participants: American and German patients and healthcare professionals from US and Germany	Discussion, documentation	Advance directive; end-of-life decision
2.	Htut Y (2007)[57]	In-depth (semi-structured) interview	Malaysia	Hospitals (4 outpatient clinic and 11 inpatient ward)	Elderly patients	15	Discussion, documentation	Advance care planning, advance directive
3.	Lee J (2010)[32]	Cross sectional survey	South Korea	Hospital	Patients with advanced lung cancer	30 patients (out of 124 participants). Other participants: family members, physicians, nurses	Documentation	Advance directive
4.	Chu LW (2011)[39]	Cross-sectional survey	Hong Kong	Long-term care facilities	Elders living in long-term care facility	1600	Documentation	Advance directive
5.	Ting FH (2011)[37]	Cross sectional survey	Hong Kong	Hospital (inpatient, acute wards)	Elderly in-patients with chronic diseases	219	Documentation	Advance directive
6.	Ivo K (2012)[24]	Cross-sectional survey	South Korea, Japan, China	Hospital	Seriously-ill patients with cancer	205: 91 (South Korea); 52 (Japan); 62 (China)	Documentation	Advance directive
7.	Keam B (2013)[34]	Cross-sectional survey	South Korea	Hospital	Patients with cancer (any stage)	1242 (out of 3840). Other participants: family caregivers, oncologists, and general public	Documentation	Advance directive

No.	First author (year)	Study Design	Country or region	Setting	Subjects	Number of participants	Element of ACP being studied	Term related to ACP studied
8.	Ni P (2014)[56]	Cross-sectional survey	China	Long-term care facilities	Elders living in long-term care facility	467	Documentation	Advance directive
9.	Cheong K (2015)[44]	Mixed method	Singapore	Hospital	Patients with early cognitive impairment	93	Discussion	Advance care planning
10.	Park SY (2015)[31]	Cross sectional survey	South Korea	Long-term care facilities	Elders living in long-term care facility	150 (out of 300). Other participants: elders living at home	Discussion, documentation	DNR decision, DNR order
11.	Hing Wong A (2016)[54]	Pre and post-test survey	Malaysia	Hospital	Patients on routine hemodialysis	58	Discussion	Advance care planning
12.	Hui EC (2016)[38]	Cross-sectional survey	Hong Kong	Hospital	Patients with solid cancer (any stage)	288 (149 palliative and 139 non-palliative)	Documentation	Advance directive
13.	Lee HTS (2016)[50]	In-depth interview	Taiwan	Long-term care facilities	Elders living in long-term care facility	11	Documentation	DNR directive
14.	Park J (2016)[35]	Cross-sectional survey	South Korea	Community centers and nursing homes	Elders living in long-term care facility	156	Documentation	Advance directive
15.	Zhang Q (2016)[52]	Cross sectional survey	China	Cancer center hospital (inpatient wards)	In-patients with solid cancer (any stage)	209 (out of 424). Other participants: family caregivers	Documentation	Advance directive
16.	Zheng RJ (2016)[51]	Cross-sectional survey	China	Cancer center hospitals (inpatient wards)	In-patients with solid cancer (any stage)	526	Documentation	Advance directive

No.	First author (year)	Study Design	Country or region	Setting	Subjects	Number of participants	Element of ACP being studied	Term related to ACP studied
17.	Hirakawa Y (2017)[25]	Semi-structured interview	Japan	Home care support	Elders requiring home care services	102	Discussion	End-of-life care decision
18.	Koh SJ (2017)[29]	Focus group interview	South Korea	General hospital (inpatient wards)	Elderly inpatients with major diseases	12 (out of 28). Other participants: family caregivers	Discussion, documentation	Advance care planning, advance directive
19.	Lo TJ (2017)[43]	Mixed-method	Singapore	Hospital	Patients with early cognitive impairment	158	Discussion	Advance care planning
20.	Sung HC (2017)[49]	Quasi-experimental	Taiwan	Long-term care facilities	Elders living in long-term care facility	57 (29 experimental and 28 control group)	Discussion	Advance care planning
21.	Hou XT (2018)[55]	Cross sectional survey	China	Cancer hospital	Patients with advanced cancer	264	Discussion	Advance care planning
22.	Menon S, 2018[42(a)]	Focus group and individual in-depth interview	Singapore	Geriatrics or family medicine institutions	Patients with life-limiting illness	15 (out of 61). Other participants: family caregivers and healthcare professionals	Discussion	Advance care planning
23.	An HJ (2019)[30]	Cross sectional survey	South Korea	General hospitals	Patients with terminal cancer	336	Discussion, documentation	End-of-life decision-making, POLST
24.	Chan CWH (2019)[40]	Semi-structured interview	Hong Kong	Hospital	Patients with life-limiting illness	24 (out of 96). Other participants: healthcare professionals, patient's family members, volunteers	Discussion, documentation	Advance directive, AD-decision-making, AD discussion
25.	Cheng HB (2019)[36]	Mixed-method	Hong Kong	Palliative care clinic	Patients with non-cancer life limiting illness	119	Discussion, documentation	Advance directive, end-of-life decision-making

No.	First author (year)	Study Design	Country or region	Setting	Subjects	Number of participants	Element of ACP being studied	Term related to ACP studied
26.	Kim JW (2019)[28]	Cross-sectional survey	South Korea	Cancer center hospital	Patients with advanced solid cancer	101	Documentation	POLST
27.	Lin CP (2019)[47]	Semi-structured qualitative interview	Taiwan	Oncology unit	Patients with advanced cancer	15 (out of 45). Other participants: family caregivers, healthcare professionals	Discussion	Advance care planning
28.	Lee HTS (2019)[48]	Qualitative study (Action research)	Taiwan	Long-term care facilities	Elders living in long-term care facility	10 (out of 34). Other participants: family, medical staff	Discussion	Advance care planning
29.	Park HY (2019)[33]	Cross sectional survey	South Korea	Hospital (outpatient clinic)	Patients with cancer (any stage)	1001 (out of 4176). Other participants: family caregivers, physicians, and general public	Discussion, documentation	Advance care planning, advance directive
30.	Cheung JTK (2020)[41]	Focus group and individual semi-structured interview	Hong Kong	Palliative day care center	Patients with serious illness	17 (out of 30). Other participants: family caregivers	Discussion	Advance care planning
31.	Chou HH (2020)[46]	Cross-sectional survey	Taiwan	Hospital (Neurology clinic)	Patients with early cognitive impairment	260	Documentation	Advance directive
32.	Feng C (2020)[53]	Cross-sectional survey	China	Hospital outpatient clinic	Patients with lung cancer (any stage)	148 (out of 297). Other participants: family caregivers	Documentation	Advance directive
33.	Jiao NX (2020)[58]	Semi-structured interview	Malaysia	Long-term care facility	Elders living in long-term care facility	13	Discussion	End-of-life communication
34.	Kizawa Y(2020)[23]	Pilot randomized controlled trial	Japan	No restriction on the setting	Elderly patients with chronic disease	220	Discussion	Advance care planning

No.	First author (year)	Study Design	Country or region	Setting	Subjects	Number of participants	Element of ACP being studied	Term related to ACP studied
35.	Lin CP (2020)[45]	Mixed-method	Taiwan	Hospital	Patients with advanced cancer	10 (out of 29). Other participants: family members and healthcare professionals	Discussion	Advance care planning
36.	Yoo SH (2020)[27]	Prospective cohort study	South Korea	Academic hospitals	Patients with advanced solid and/or hematologic cancer	150: (out of 251). Other participants: family caregivers	Discussion, documentation	Advance directive, POLST

ACP: advance care planning; AD: advance directive; DNR: do-not-resuscitate; POLST: physician orders for life-sustaining treatment

[(a)]Studies including participants other than patients

[(b)]Multi-country study: US, Germany, Japan

Appendix 3. Quality assessment scores for included quantitative and mixed-method studies

	Author (Reference)	Objective	Study design	Subject selection	Subject characteristics	Random allocation	Blinding of investigator	Blinding of subjects	Outcome measures	Sample size	Analytic method	Estimate of variance	Control for confounding	Result	Conclusion	Total Sum	Summary score	Quality
1.	Voltz R[26]	2	1	1	1	N/A	N/A	N/A	1	1	1	1	N/A	1	0	10	0.50	Low
2.	Lee J[32]	2	2	1	2	N/A	N/A	N/A	2	2	1	1	0	2	2	17	0.77	Good
3.	Chu LW[39]	2	2	2	2	N/A	N/A	N/A	2	2	2	2	N/A	2	2	20	1.00	Strong
4.	Ting FH[37]	2	2	2	2	N/A	N/A	N/A	2	2	1	2	N/A	2	2	19	0.95	Strong
5.	Ivo K[24]	2	2	1	1	N/A	N/A	N/A	2	1	1	1	0	2	2	13	0.59	Adequate
6.	Keam B[34]	2	2	2	1	N/A	N/A	N/A	2	2	2	2	2	2	2	21	0.95	Strong
7.	Ni P[56]	2	2	2	2	N/A	N/A	N/A	2	1	2	2	N/A	2	2	19	0.95	Strong
8.	Cheong K[44]*	2	1	1	2	N/A	N/A	N/A	1	0	1	1	0	1	1	11	0.50	Adequate[a]
9.	Park SY[31]	2	2	2	2	N/A	N/A	N/A	1	2	2	0	0	2	1	16	0.73	Good
10.	Hing Wong A[54]	2	1	1	1	N/A	N/A	N/A	1	0	2	0	N/A	2	2	12	0.60	Adequate
11.	Hui EC[38]	1	1	1	1	N/A	N/A	N/A	1	1	2	1	0	2	1	12	0.55	Adequate
12.	Park J[35]	2	2	1	1	N/A	N/A	N/A	1	1	2	2	N/A	1	2	15	0.75	Good
13.	Zhang Q[52]	2	2	1	2	N/A	N/A	N/A	2	0	2	2	N/A	1	2	16	0.80	Good
14.	Zheng RJ[51]	2	2	2	2	N/A	N/A	N/A	1	0	2	2	N/A	1	2	16	0.80	Good
15.	Lo TJ[43]*	2	1	2	2	N/A	N/A	N/A	1	1	1	1	N/A	1	2	14	0.68	Adequate[a]

	Author (Reference)	Objective	Study design	Subject selection	Subject characteristics	Random allocation	Blinding of investigator	Blinding of subjects	Outcome measures	Sample size	Analytic method	Estimate of variance	Control for confounding	Result	Conclusion	Total Sum	Summary score	Quality
16.	Sung HC[49]	2	2	1	2	0	0	N/A	2	1	2	1	0	2	2	17	0.65	Adequate
17.	Hou XT[55]	2	1	1	2	N/A	N/A	N/A	1	0	2	1	N/A	2	1	13	0.75	Good
18.	An HJ[30]	2	1	1	2	N/A	N/A	N/A	2	1	1	0	N/A	1	1	12	0.60	Adequate
19.	Cheng HB[36]	2	1	1	1	N/A	N/A	N/A	2	1	2	1	N/A	2	2	16	0.8	Good[a]
20.	Kim JW[28]	2	2	1	2	N/A	N/A	N/A	1	1	2	1	0	2	2	16	0.73	Good
21.	Park HY[33]	2	2	2	2	N/A	N/A	N/A	2	2	2	2	2	2	2	22	1.00	Strong
22.	Chou HH[46]	2	2	1	1	N/A	N/A	N/A	1	1	1	1	1	1	1	13	0.50	Low
23.	Feng C[53]	1	2	0	2	N/A	N/A	N/A	1	1	2	0	N/A	1	2	12	0.6	Adequate
24.	Kizawa Y[23]	2	2	2	1	1	1	0	1	2	2	2	1	2	2	21	0.75	Good
25.	Lin CP[45]*	2	2	2	1	N/A	N/A	N/A	2	1	2	2	N/A	2	2	18	0.93	Strong[a]
26.	Yoo SH[27]	2	2	1	2	N/A	N/A	N/A	1	1	2	2	N/A	2	2	17	0.85	Strong

N/A: not applicable.
[a]Mixed-method study: summary score is the sum of quality assessment scores for qualitative and quantitative divided by two.

Appendix 4. Quality assessment scores for included qualitative and mixed-method studies

	Author (Reference)	Objective	Study design	Context of the study	Theoretical framework	Sampling strategy	Data collection	Data analysis	Verification procedure	Result and conclusion	Reflexivity of the account	Total sum	Summary Score	Quality
1.	Htut Y[57]	2	2	2	0	1	2	1	2	2	0	14	0.70	Adequate
2.	Cheong K[44]*	2	1	2	1	2	1	2	2	2	0	15	0.75	Adequate[a]
3.	Lee HTS[50]	2	2	2	1	2	2	2	2	2	2	19	0.95	Strong
4.	Hirakawa Y[25]	2	2	2	1	1	2	2	2	2	0	16	0.80	Good
5.	Koh SJ[29]	1	2	2	1	1	2	2	0	1	0	12	0.60	Adequate
6.	Lo TJ[43]*	2	1	2	1	1	1	2	2	1	0	13	0.68	Adequate[a]
7.	Menon S[42]	2	2	2	1	2	2	2	2	2	0	17	0.85	Strong
8.	Chan CWH[40]	2	2	1	1	2	0	1	0	2	0	11	0.55	Adequate
9.	Cheng HB[36]	2	1	2	0	1	2	0	0	2	0	10	0.8	Good[a]
10.	Lin CP[47]	2	2	2	1	2	2	2	2	2	1	18	0.90	Strong
11.	Lee HTS[48]	2	2	2	2	2	2	2	2	2	0	18	0.90	Strong
12.	Cheung JTK[41]	2	1	2	1	2	2	2	2	2	0	16	0.80	Good
13.	Jiao NX[58]	2	2	2	0	1	1	1	2	2	0	13	0.65	Adequate
14.	Lin CP[45]*	2	2	2	2	1	2	2	2	2	2	19	0.95	Strong[a]

N/A: not applicable.
[a]Mixed-method study: summary score is the sum of quality assessment scores for qualitative and quantitative divided by two.

Appendix 5. Asian patients' agreement with the importance of advance directive

No	First author	Year	Country/Region	Type of patients	Asian patients' perspectives on their agreement with importance of advance directives	Percentage
1.	Ivo K[24]	2012	South Korea, China, Japan	Seriously-ill patients with cancer	Agree with the importance of AD	South Korea: 85%; China: 80%; Japan: 96%
2.	Park SY[31]	2015	South Korea	Elders living in long-term care facility	Agree with the potential importance of DNR order	87%
3.	Keam B[34]	2013	South Korean	Patients with cancer (any stage)	Agree with the importance of AD	93%
4.	Hing Wong A[54]	2016	Malaysia	Patients on routine hemodialysis	Agree with the importance of AD	75%
5.	Zhang Q[52]	2016	China	Patients with cancer	Agree with the importance of AD<br>Disagree with the importance of AD	74%<br>26%
6.	Zheng RJ[51]	2016	China	In-patients with solid cancer (any stage)	Agree with the importance of AD<br>Disagree with the importance of AD	22%<br>78%
7.	Chou HH[46]	2020	Taiwan	Patients with early cognitive impairment	Agree with the importance of AD	77%

AD: advance directive; DNR: do-not-resuscitate.

Appendix 6. Patients' perspectives on the decision maker in advance care planning

No	First author	Year	Country	Type of patient	Patients' perspectives on decision maker in ACP	Major findings
1.	Voltz R[26]	1998	Japan	Patients with terminal illness	Family	29%
2.	Lee J[32]	2010	South Korea	Patients with advanced lung cancer	a) Patient<br>b) Patient and family<br>c) Family	a) 27%<br>b) 63%<br>c) 10%
3.	Ting FH[37]	2011	Hong Kong	Elderly in-patients with chronic diseases	a) Patient<br>a) Patient, family, and physician	a) 55%<br>a) 14%
4.	Ivo K[24]	2012	South Korea, China, Japan	Seriously-ill patients with cancer	b) Patient<br>c) Patient and family<br>d) Patient and physician<br>e) Patient, family, and physician<br>f) Family<br>g) Physician	b) 27% (South Korea); 26% (China); 33% (Japan)<br>c) 40% (South Korea); 37% (China); 49% (Japan)<br>d) 6% (South Korea); 11% (China); 8% (Japan)<br>e) 21% (South Korea); 0 (China); 4% (Japan)<br>f) 3% (South Korea); 18% (China); 4% (Japan)<br>g) 2% (South Korea); 8% (China); 2% (Japan)
5.	Park SY[31]	2015	South Korea	Elders living in a long-term care facility	a) Patient<br>b) Patient and family<br>c) Family and physician<br>d) Other	a) 39%<br>b) 32%<br>c) 25%<br>d) 4%
6.	Hui EC[38]	2017	Hong Kong	Patients with solid cancer (any stage)	a) Patient<br>b) Patient and family<br>c) Patient and physician<br>d) Patient, family, and physician<br>e) Family and physician<br>f) Physician<br>g) Family	a) 21%<br>b) 13%<br>c) 3%<br>d) 55%<br>e) 3%<br>f) 4%<br>g) 1%

7.	Kim JW[28]	2020	South Korea	Patients with advanced solid cancer	a) Patient<br>b) Family<br>c) Physician	a) 51%<br>b) 21%<br>c) 18%

ACP: advance care planning.

Appendix 7. Underlying motives for patients' willingness or unwillingness to engage in ACP (Quantitative data)

No	First author	Year	Country	Type of patient	Motives for patient's willingness to engage in ACP	Percentage
1.	Ting FH[37]	2011	Hong Kong	Elderly in-patients with chronic diseases	a) Patients' belief that ACP would ensure comfortable end-of-life<br>b) Patients' belief that ACP would avoid causing burden to the family<br>c) Patients' belief that ACP would ensure their wishes will be respected<br>d) Patients' belief that ACP would prevent conflict between family members<br>e) Patients' experience with the death of a relative/friend<br>f) Patients' belief that ACP would avoid causing burden to the society<br>g) Patients' belief that quality of life is more important than the length of life<br>h) Patients' religious beliefs	a) 71%<br>b) 39%<br>c) 35%<br>d) 14%<br>e) 9%<br>f) 8%<br>g) 8%<br>h) 4%
2.	Ni P[56]	2014	China	Elders living in long-term care facility	a) Patients' wish to make AD when they are still cognitively intact<br>b) Patients' wish that ACP would help the family understand their wishes at an early stage	a) 44%<br>b) 39%
3.	Park J[35]	2016	South Korea	Elders living in long-term care facility	a) Patients wish to avoid causing a burden to the family with end-of-life decisions<br>b) Patients' wish to decide for themselves<br>c) Patients' belief of the possibility of incapacity due to their illness	a) 61%<br>b) 61%<br>c) 54%
4.	Kim JW[28]	2020	South Korea	Patients with advanced solid cancer	a) Patients' wish to exercise self-determination<br>b) Patients' wish to follow physician's recommendation<br>c) Patients' belief that ACP would prevent them from suffering due to meaningless treatment<br>d) Patients' wish to ease the economic burden on the family	a) 39%<br>b) 35%<br>c) 26%<br>d) 13%

No	First author	Year	Country	Type of patient	Motives for patient's unwillingness to engage in ACP	Percentage

1	Ting FH[37]	2011	Hong Kong	Elderly in-patients with chronic diseases	a) Patients’ wish to entrust decision-making to the relatives<br>b) Patients’ belief to let nature take its course<br>c) Patients’ belief that there is no need to think about drafting an AD now<br>d) Patients’ wish to entrust decision-making to the physicians<br>e) Patients’ belief that they may want to change their decision later<br>f) Patients’ belief that they are not familiar with the concept of AD<br>g) Patients’ religious beliefs	a) 39%<br>b) 25%<br>c) 23%<br>d) 13%<br>e) 3%<br>f) 3%<br>g) 2%
2	Ni P[56]	2014	China	Elders living in a long-term care facility	a) Patients’ lack of awareness about AD<br>b) Patients’ wish to entrust decision-making to the family<br>c) Patients’ belief that ACP is not useful even when it is completed<br>d) Patients’ belief that it is too early for ACP	a) 66%<br>b) 23%<br>c) 6%<br>d) 3%
3	Park J[35]	2016	South Korea	Elders living in a long-term care facility	a) Patients’ wish to entrust decision-making to the family<br>b) Patients’ wish to entrust decision-making to the physician<br>c) Patients’ belief that ACP is not necessary for their current age<br>d) Patients’ lack of knowledge regarding ADs	a) 67%<br>b) 60%<br>c) 44%<br>d) 30%
4	Hui EC[38]	2017	Hong Kong	Patients with solid cancer (any stage)	a) Patients’ belief that it is difficult to make such medical decisions before it happens<br>b) Patients’ belief that they need more information and time for ACP<br>c) Patients’ wish to entrust decision-making to the family	a) 72%<br>b) 55%<br>c) 53%
5	Hou XT[55]	2018	China	Patients with advanced cancer	a) Patients’ wish to entrust decision-making to the relatives<br>b) Patients’ wish to entrust decision-making to their physicians<br>c) Patients’ belief that talking about ACP would make their relatives sad<br>d) Patients’ belief that talking about ACP would make them sad<br>e) Patients’ belief that they do not have enough information needed for decision-making<br>f) Patients’ belief that they have a lack of understanding of how to approach end-of-life communication	a) 31%<br>b) 29%<br>c) 23%<br>d) 19%<br>e) 19%<br>f) 17%
6	An HJ[30]	2019	South Korea	Patients with terminal cancer	a) Patients’ belief that they have lack knowledge about AD<br>b) Patients’ belief that drafting AD would mean giving up or result in being abandoned by the physicians<br>c) Patients’ belief that signing AD would lead to bad things<br>d) Patients’ discomfort in talking about death<br>e) Patients wish to entrust decision-making to the family<br>f) Patients’ wish to entrust decision-making to the physician<br>g) Patients’ lack of understanding/denial of their prognosis	a) 65%<br>b) 30%<br>c) 44%<br>d) 30%<br>e) 44%<br>f) 35%<br>g) 14%

7	Park HY[33]	2019	South Korea	Patients with cancer (any stage)	a) Patients' belief that they may change their mind in the future when facing the real situation<br>b) Patients belief that ACP would cause psychological discomfort<br>c) Patients' belief that their wish would not be respected<br>d) Patients' belief that they have a lack of understanding about AD<br>e) Patients' belief that the family will make the best decision on their behalf	a) 22%<br>b) 22%<br>c) 21%<br>d) 13%<br>e) 12%
8	Kim JW[28]	2020	South Korea	Patients with advanced solid cancer	a) Patients' belief that they need to further discuss with the family<br>b) Patients' belief that they require more time prior to completion of ACP documentation<br>c) Patients' belief it is too early for ACP<br>d) Patients' belief that they have a lack of understanding of local policy	a) 38%<br>b) 28%<br>c) 21%<br>d) 17%

ACP: advance care planning; AD: advance directive.

Appendix 8. Motives for patients' willingness or unwillingness to engage in advance care planning (Qualitative data)

No	Motives for patient's willingness to engage in ACP	Related findings or quotes	Number of studies (References)
1.	Patients' belief that ACP would promote autonomy	*"I want to manage my life on my own until the end"*	5 ([25, 42, 44, 57, 58])
2.	Patients' wish to have comfort near the end of their life	*"Don't wake me up when I die. Just imagine when you wake up, you might find yourself paralyze. What's the point of living when paralyzed? You're only surviving for your friends and love ones. I don't want that, that's suffering"*	3 ([40, 57, 58])
3.	Patients' wish to avoid being a burden to their family	*"If I develop severe dementia, I would like to be institutionalized to avoid becoming a burden on my wife"*	4 ([25, 42, 44, 57])
4.	Patients' belief that ACP would create a connection with the family	Patients thought they could get closer to their families, made it easier for their caregivers to look after them, and gave them the opportunity to fulfill their wishes	1 ([42])
5.	Patients' experience with ACP	*"Finally ... it is a form of relief. At last, I can speak up openly. Thank you for giving me this chance. I suppose it was not that hard to open up about this topic. I want to find some time to discuss this with my family"*	2 ([45, 58])
**No**	**Motives for patient's unwillingness to engage in ACP**	**Related findings or quotes**	**Number of studies (References)**

1.	Patients' lack of illness understanding	The majority of them did not seem to understand the gravity or seriousness of their illness. For instance, a fairly educated gentleman with severe chronic obstructive airway disease, who was recently admitted to the high dependency unit, stated that he was very hopeful and very optimistic of making a complete recovery	4 ([25, 36, 44, 57])
2.	Incomplete understanding/lack of awareness regarding ACP	Lack of awareness of early discussion A patient believed that she could not participate in ACP discussion, because she would not be able to do so at the end-of-life stage<br><br>*"At that moment (end of life) I am already...how can I make a decision? I will leave it to my family for sure"*	8 ([41-44, 48, 50, 57, 58])
3.	Patients' lack of understanding of ACP relevance for planning beyond financial arrangements	Patients who claimed to "have no property" to plan for and only "relied on children's monthly contribution" for their living expenses also did not complete advance care plans as they failed to appreciate the need for ACP beyond financial concerns<br><br>Patient (53) thought that *"it is important to complete ACP but thinks he does not have much assets to worry",* while patient (51) reported that *"she has little property and finances, so no immediate need [for ACP]"*	2 ([43, 44])
4.	Patient's concern that they would feel uncomfortable discussing end-of-life issues/loss of hope	Patients were concerned that discussions about end-of-life matters may cause them to become sad or fearful<br><br>*"When I was healthy, I sometimes thought about it, but I don't think it is necessary, and I feel sad…. I just don't want to talk about it ever since I became sick"*	3 ([29, 41, 42])
5.	Patients' concern that ACP would cause distress for family members	Patients were concerned that the ACP may burden family members who had problems of their own to manage<br><br>*"Death is just death…why should I worry right now? If I talk to my family about end-of-life care…it will seem that I am threatening my family and making them feel sorrow…I don't want to do this"*	5 ([41, 42, 48, 50, 58])
6.	Patients' concern that ACP would cause conflict within their family members	Patient (3) shared that "she is wary about doing (advance directive) as she is worried that it will cause conflict between her two sons", whereas patient (75)	3 ([44, 50, 58])

		expressed "she does not want her children to be unhappy that she is 'playing favorites' by appointing certain children as 'done'"	
7.	Patient's belief that discussing end-of-life would bring bad luck (*taboo*)	Not wanting to cause problems, no participant wanted to discuss death or end-of-life care with family. Most of the participants and their children believed that discussing end-of-life-care-related concerns would bring them bad luck<br><br>*"No, [frown] in fact this kind of issue (end-of-life planning) shouldn't be discussed openly as it is a taboo subject and not something we can discuss openly. I can't tell you why, but like I said just now, you can't simply open-up and discuss this thing. You might not know, something bad might happen after the discussion"*	2 ([50, 58])
8.	Patients inability to appreciate what intent of ACP	*"It is unnecessary and ridiculous for me to think about whether to receive CPR or not…or…what kind of care I want to have at the end of life right now…the only thing I want to do right now is to live here happily and smoothly"*	2 ([43, 50])
9.	Patients' doubted the effectiveness of ACP in conveying their wishes	Patient (54) reported he was "not keen to consider ACP as he has reservations and lack of trust over wife and children's abilities to follow his wishes"	1 ([44])
10.	Patients' belief that HCPs do not advocate ACP	*"The consultant told me if I received the treatment then my life could be extended for 2 months. If not, my life would be shortened for 2 months … At that time, I didn't want to receive (the treatment). But eventually I received the treatment three times. Later, he said I could continue to undergo the treatment. The more treatments I received, the more sluggish I was. I asked whether I could quit. The consultant questioned me, "Really?"… And in the next consultation, the doctor told me, I could keep receiving treatment in view of my condition … He asked 'How about getting the treatment again?'"*	2 ([41, 43])
11.	Patients' belief that family does not support their engagement in ACP	*"In my experience, you are concerned mainly about your family's opinions [when you make a decision] rather than your own opinions at the end of life. (PT15: 57 y/o lung cancer female)"*	3 ([43, 44, 47])
12.	Patients' wish to seek harmony with the mandate of nature	Furthermore, they expressed that death is a natural event and that a human must seek harmony with nature rather than try to change it<br><br>*"I don't want to think or talk too much about end-of-life care…just let it happen naturally…everything must follow the mandate of nature… just as that*	1 ([50]

		tree outside the window accepts its situation from nature without questioning"	
13.	Patients' belief in providence	*"Let me tell you…when you will die and how to die… these things have been decided already by what you have done in present and past lives…it is really complex…we may never be able to understand the language of providence"*	7 ([41, 44, 48, 50, 57, 58])
14.	Patients' concern that their decisions may change in the future	*"I don't want to discuss or decide in advance because nobody knows what will happen in the future. New treatment could be introduced in the future, and I may change my mind even…."*	2 ([29, 42])
15.	Patients' concern of difficulty in planning for the unknown/unpredictable disease course	*"I have no idea about things related to end-of-life care or signing DNR papers…they are too complex for me to make decisions by myself without their [my children's] permission…so, please ask my children…they are smarter than I am, and they can make any decision for me by themselves"*	4 ([25, 41, 45, 50])
16.	Patients considered ACP irrelevant due to their socioeconomic dependency	She leaves long-term planning to her niece's family as she does not have the resources to plan for herself and trusts them to make plans for her	4 ([25, 43, 44, 58])
17.	Patients' belief of limited options available for them in the future care	*"I think I would have no choice but to be institutionalized in the future, just as my sister was"*	1 ([25])
18.	Patients' belief that limited care continuity hampers ACP	Under the healthcare system of Hong Kong, patients usually are seen by different doctors across visits in the same clinical settings. They could hardly develop a long term relationship and have continual communication with the same doctors. This might also prevent continual ACP discussion *"Consultations are delivered by different doctors. It is not the same person every time"*	1 ([41])
19.	Patients' belief that time constraint from HCPs side hampers ACP	*"I want to know the diagnosis and prognosis. I want to know what will happen if the condition keeps worsening. But doctors (in acute setting) were really too busy. He (doctor) talked with me in the corridor. That's depressing."*	1 ([41])
20.	Patients' belief that HCPs lack the communication skills and empathy for ACP	*"A doctor yelled "if you don't receive the treatment you will die" (in an oncology inpatient setting). From the perspective of patients, such words will make us feel down. So I think doctors, I don't know the reasons, but I think as a professional, the best way to communicate is not to say those words to a person in need … Sometimes their words are discouraging."*	1 ([41])
21.	Patients' wish to entrust decision-making to family members	*"Ideally, these end-of-life decisions should be left with your family, because they know your wishes and can fulfil them in the way you want when your life*	9 ([25, 36, 41-44, 50, 57, 58])

		ends. [Laugh] Die in your own way ... You see, we can prepare and cope with the cycle of life, from birth, growing old and sickness, but when it comes to death how many of us are able to die in our own way? [Frown] No .. . your body might belong to you, but how you die and what happens next is for others, especially your family"	
22.	Patients' belief that the physicians would "do what is right"	*"Doctors are professionals…if doctors think I can't be saved…just let me die soon…if doctors think I will have the chance to live longer…let me have CPR…I have told my children that they need to trust doctors' ability and follow their orders without question"*	4 ([41, 50, 57, 58])
23.	Patients' belief that informal planning would suffice	*"I already talked to my children about how I want to be treated and to spend the rest of my life."*	3 ([29, 44, 57])
24.	Patients' belief that it is too early to engage in ACP	*"Because…I am healthy presently and too young to think about issues related to death or end-of-life care… would you please not talk about these issues until I am more than 70 years old?"*	2 ([25, 50])

ACP: advance care planning; CPR: cardiopulmonary resuscitation; DNR: do-not-resuscitate; HCPs: healthcare professionals.
